# Atherogenic index of plasma and lower estimated glomerular filtration rate in IgA nephropathy

**DOI:** 10.1186/s12944-026-02896-4

**Published:** 2026-02-18

**Authors:** Ricong Xu, Abdul Rashid Qureshi, Mohamed E. Suliman, Nanbo Zhu, Hong Xu, Yuna Chen, Anni Zhong, Qijun Wan, Bengt Lindholm

**Affiliations:** 1 Department of Nephrology, Shenzhen Second People’s Hospital, The First Affiliated Hospital of Shenzhen University, Shenzhen, Guangdong China; 2 Division of Renal Medicine, Department of Clinical Science Intervention and Technology, Karolinska Institutet, Stockholm, Sweden; 3 Department of Neurobiology, Care Sciences and Society, Karolinska Institutet, Stockholm, Sweden

**Keywords:** IgA nephropathy, Atherogenic index of plasma, Chronic kidney disease, Dyslipidemia, Body mass index, Hyperuricemia

## Abstract

**Background:**

IgA nephropathy (IgAN) demonstrates substantial progression to end-stage kidney disease, yet metabolic risk factors remain underexplored. The atherogenic index of plasma (AIP), calculated as log(triglycerides/HDL-cholesterol), integrates pro-atherogenic and anti-atherogenic lipid components, but its association with renal function in IgAN is unclear.

**Methods:**

This cross-sectional study analyzed 1186 Chinese patients with biopsy-proven primary IgAN. AIP was standardized as Z-scores for analysis. Primary outcome was estimated glomerular filtration rate (eGFR). Secondary outcomes included eGFR < 60 mL/min/1.73 m² and proteinuria. Multivariable linear and logistic regression models assessed associations with progressive adjustments. Subgroup analyses evaluated effect modification.

**Results:**

Median age was 34 years with 48% males. Each standard deviation increase in AIP was associated with 2.08 mL/min/1.73 m² lower eGFR (95% CI: -3.48, -0.68; *p* = 0.004) and 33% higher odds of eGFR < 60 mL/min/1.73 m² (OR: 1.33; 95% CI: 1.07, 1.67; *p* = 0.012) in fully adjusted models. Patients in the highest versus lowest AIP tertile had 4.73 mL/min/1.73 m² lower eGFR (*p* = 0.005) and 76% higher odds of eGFR < 60 mL/min/1.73 m² (*p* = 0.034). AIP was associated with higher proteinuria in patients with eGFR ≥ 60 mL/min/1.73 m² (β: 141.63 mg/24 h per SD; 95% CI: 33.75, 249.51; *p* = 0.01). Significant effect modification occurred by BMI (*p*-interaction = 0.032) and hyperuricemia (*p*-interaction = 0.030), with stronger associations in patients with BMI < 23 kg/m² and without hyperuricemia.

**Conclusion:**

Higher AIP independently associates with lower eGFR and higher proteinuria at diagnosis in IgAN patients, particularly in specific metabolic subgroups. Longitudinal studies are needed to determine whether AIP has prognostic value for renal outcomes.

**Supplementary Information:**

The online version contains supplementary material available at 10.1186/s12944-026-02896-4.

## Background

IgA nephropathy (IgAN) represents the most prevalent primary glomerulonephritis globally, accounting for approximately 54.3% of all primary glomerulonephritis cases in China [1]. Despite advances in understanding its pathogenesis, long-term outcomes remain suboptimal, with recent cohort studies demonstrating that 46% of patients progress to end-stage renal disease within 10 years and 71% by 20 years after diagnosis [2]. While traditional risk factors including proteinuria, hypertension, and histologic lesions are well-established [3], the contribution of metabolic factors, particularly dyslipidemia, remains inadequately characterized.

Chronic kidney disease (CKD)-associated dyslipidemia presents a distinctive pattern characterized by elevated triglycerides (TG), reduced high-density lipoprotein cholesterol (HDL-C), and qualitative alterations in lipoprotein composition [4]. These alterations promote renal injury through multiple mechanisms including lipotoxicity-induced mitochondrial dysfunction, oxidative stress, and inflammatory cascade activation [5–7]. Notably, both the 2014 and 2024 Kidney Disease: Improving Global Outcomes (KDIGO) guidelines recognize CKD-specific dyslipidemia but do not establish TG reduction or HDL-C elevation as primary therapeutic targets, focusing instead on statin-based cardiovascular risk reduction [8, 9]. This therapeutic gap underscores the need for refined biomarkers that better capture the complex interplay between atherogenic and anti-atherogenic lipid components.

The atherogenic index of plasma (AIP), calculated as log[TG/HDL-C], has emerged as a comprehensive biomarker that mathematically integrates the pro-atherogenic potential of elevated TG with the protective effects of HDL-C [10]. By utilizing a logarithmic transformation, AIP provides superior cardiovascular risk assessment compared to individual lipid parameters [11]. Recent evidence extends AIP’s clinical utility to kidney function assessment. In Chinese populations, the TG/HDL-C ratio demonstrated superior predictive value for albuminuria compared to conventional lipid parameters [12, 13]. Among diabetic patients, each unit increase in AIP conferred a 1.93-fold higher risk of diabetic kidney disease [14]. Furthermore, elevated TG/HDL-C ratio independently predicted renal function decline regardless of baseline kidney function [15].

However, existing studies have predominantly focused on diabetic or general populations, leaving the relationship between AIP and renal function in specific glomerular diseases poorly characterized. Given IgAN’s distinct pathophysiology involving mesangial IgA deposition, complement activation, and inflammatory cascades [16], the association between AIP and renal function in this population warrants specific investigation. Furthermore, how patient characteristics such as sex, nutritional status and metabolic alterations might modify this relationship remains unexplored.

Therefore, this study aimed to investigate the cross-sectional association between AIP and renal function at diagnosis in a population of 1186 Chinese patients with IgAN. Understanding these disease-specific relationships at presentation may provide insights into the role of AIP in IgAN and inform the design of future longitudinal studies to assess the prognostic value of AIP in this prevalent glomerulopathy.

## Methods

### Study design and population

This cross-sectional study analyzed data from patients with biopsy-proven primary IgAN recorded in the IgAN Database at Shenzhen Second People’s Hospital, Shenzhen, China, between December 1, 2010, and April 30, 2025. The study protocol was approved by the Medical Ethics Committee of Shenzhen Second People’s Hospital (No. 2025-633-01PJ) and conducted in accordance with the Declaration of Helsinki. Due to the retrospective nature of the study, the requirement for informed consent was waived.

**Fig. 1:**
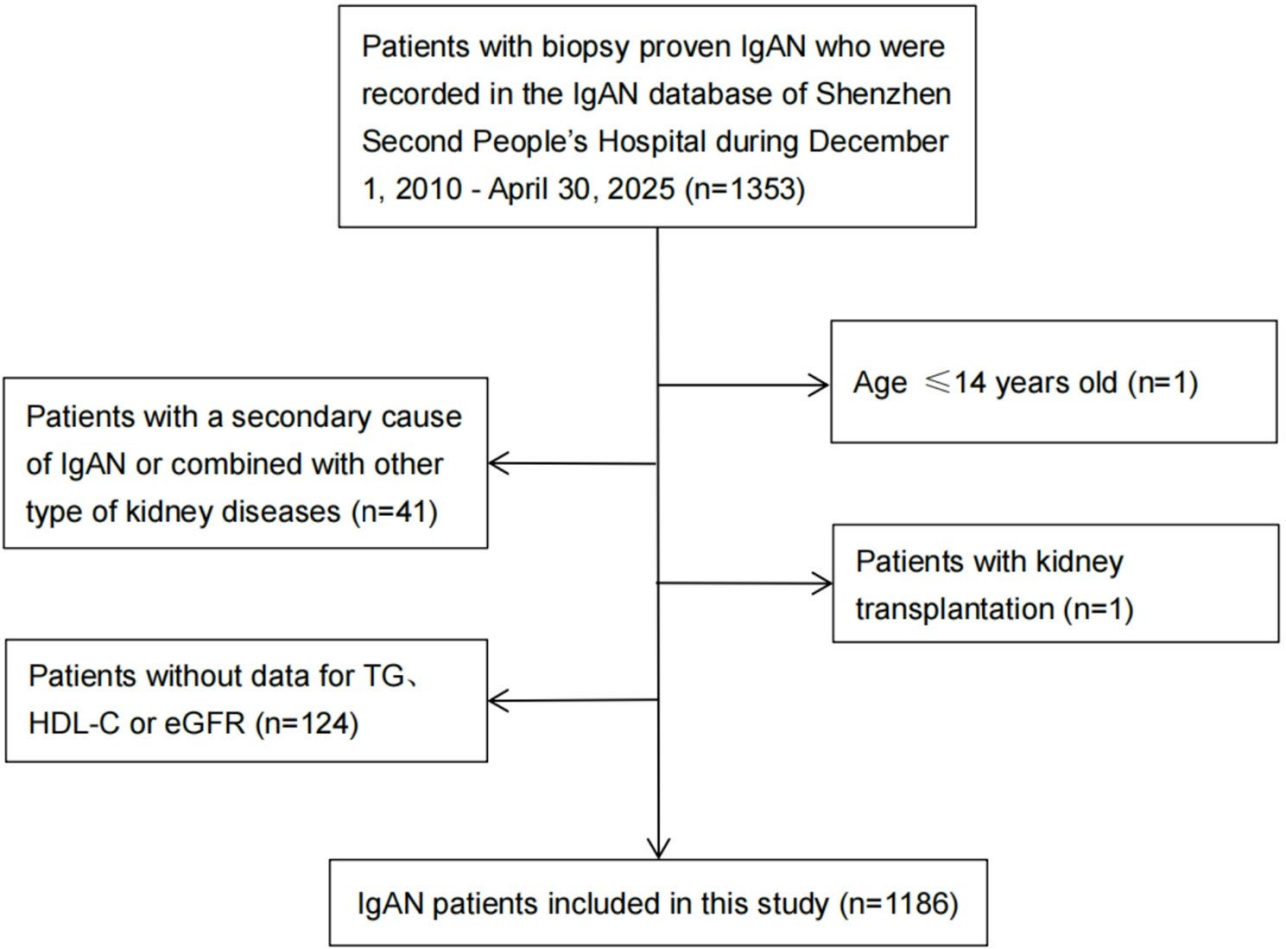
Flowchart of patients with IgA nephropathy included in the study

We initially identified 1353 patients with biopsy-proven primary IgAN through systematic review of electronic medical records and pathology reports. Exclusion criteria were: (1) age ≤ 14 years (*n* = 1); (2) secondary IgAN or concomitant kidney diseases (*n* = 41), including hepatitis B virus-associated nephropathy (*n* = 4), diabetic nephropathy (*n* = 8), anti-neutrophil cytoplasmic antibody-associated vasculitis (*n* = 2), Castleman’s disease (*n* = 1), thin basement membrane nephropathy (*n* = 4), membranous nephropathy (*n* = 3), minimal change disease (*n* = 11), malignant hypertensive nephropathy (*n* = 1), obesity-related glomerulopathy (*n* = 3), and thrombotic microangiopathy (*n* = 4); (3) receiving renal replacement therapy (*n* = 1); and (4) missing data for key variables including eGFR (*n* = 33) or lipid parameters required for AIP calculation (*n* = 91). After applying these criteria, the remaining 1186 patients were included in the final analysis (Fig. 1).

### Data collection and measurements

All data were collected at the time of renal biopsy during hospitalization. Demographic information included age, sex, and medical history (hypertension, diabetes mellitus, smoking status). Clinical measurements comprised body mass index (BMI, calculated as weight in kilograms divided by height in meters squared), systolic blood pressure (SBP), and diastolic blood pressure (DBP).

Laboratory measurements were performed using standardized methods in the hospital’s clinical laboratory. Complete blood count, serum biochemistry (including albumin, uric acid, fasting plasma glucose [FPG]), and inflammatory markers (high-sensitivity C-reactive protein [hs-CRP]) were analyzed. Lipid profiles, including TG, total cholesterol (TC), low-density lipoprotein cholesterol (LDL-C), and HDL-C, were measured using standard enzymatic methods. Immunological parameters included serum immunoglobulins (IgA, IgG, IgM) and complement components (C3, C4). Twenty-four-hour urinary protein excretion was quantified by standard methods.

Prescribed medication at baseline was recorded, including renin-angiotensin system inhibitors (RASi: angiotensin-converting enzyme inhibitors or angiotensin receptor blockers), corticosteroids and/or immunosuppressants (CSs/ISs), and lipid-lowering therapy (statins).

### Variable definitions

#### Primary exposure

AIP was calculated using the formula: AIP = log(TG/HDL-C), where both TG and HDL-C were measured in mmol/L [10]. For regression analyses, AIP was standardized as Z-scores to facilitate interpretation. Participants were also stratified into tertiles based on AIP distribution for categorical analyses.

#### Study outcomes

The primary outcome was renal function assessed by eGFR calculated using the 2021 chronic kidney disease epidemiology collaboration (CKD-EPI) creatinine-based equation without race coefficient [17], treated as a continuous variable. The secondary outcomes were eGFR < 60 mL/min/1.73 m², and 24-hour proteinuria (mg).

#### Histopathological assessment

Renal biopsy specimens underwent standard processing with light microscopy, immunofluorescence, and electron microscopy. Histopathology was graded according to the revised Oxford Classification (MEST-C score) [18]: mesangial hypercellularity (M0/M1), endocapillary hypercellularity (E0/E1), segmental glomerulosclerosis (S0/S1), tubular atrophy/interstitial fibrosis (T0/T1/T2), and crescents (C0/C1/C2). Due to limited numbers, T1 (*n* = 268) and T2 (*n* = 95) were combined as T1/2 (*n* = 363), and C1(*n* = 518) and C2 (*n* = 97) as C1/2 (*n* = 615) for analyses. All biopsies were reviewed independently by two renal pathologists, with discrepancies resolved by a third senior pathologist.

### Statistical analysis

All statistical analyses were conducted using R software (version 4.5.1, http://www.R-project.org/). Continuous variables were assessed for normality using the Kolmogorov-Smirnov test (Table S1) and presented as median (interquartile range, IQR) due to non-normal distributions. Categorical variables were expressed as counts (percentages). Comparisons across AIP tertiles used Kruskal-Wallis test for continuous variables and chi-square test for categorical variables.

 Table S2 shows the degree of data incompleteness in this investigation. Missing data (BMI: *n* = 124[10.46%]; hs-CRP: *n* = 86[7.25%]; and fasting blood glucose, FBG: 74[6.24%]; other variables: <5%) were addressed using multiple imputation by chained equations (MICE) with 20 imputed datasets, assuming missing at random [19, 20]. The imputation model included all demographic, clinical, laboratory, and histopathological variables. Primary analyses used pooled estimates across imputed datasets with Rubin’s rules for variance estimation.

Multiple linear regression examined associations between AIP (as standardized Z-scores) and eGFR. Three models were constructed: Model 1 (unadjusted); Model 2 (adjusted for age, sex, smoking, BMI, diabetes, SBP, DBP); Model 3 (additionally adjusted for uric acid, proteinuria, hs-CRP, tubular atrophy/interstitial fibrosis, and statin use). For analyses of association between AIP and proteinuria, Model 3 was modified to exclude proteinuria as a covariate and include eGFR instead. Logistic regression using identical models evaluated associations with eGFR < 60 mL/min/1.73 m².

Restricted cubic spline (RCS) regression examined potential non-linear relationships between AIP (as standardized Z-scores) and the risk of eGFR < 60 mL/min/1.73 m².

To evaluate the incremental value of AIP beyond established clinical, laboratory, and histological predictors, we compared model performance with and without AIP using multiply imputed data. For eGFR as a continuous outcome, we compared models based on mean R², mean Akaike information criterion (AIC), and likelihood ratio tests across imputed datasets. For the binary outcome of eGFR<60mL/min/1.73 m², we assessed discrimination using receiver operating characteristic (ROC) curves and compared area under the curve (AUC) between models with and without AIP using DeLong’s test, with results averaged across imputations.

Subgroup analyses examined effect modification by age (< 34 vs. ≥34 years), sex, BMI (< 23 vs. ≥23 kg/m²), blood pressure (SBP < 140 vs. ≥140 mmHg; DBP < 90 vs. ≥90 mmHg), proteinuria levels, and hyperuricemia status. The age categorization was based on the median age of the study population. According to the World Health Organization classification for Asian populations, patients were categorized as underweight, normal weight, overweight, and obese using the thresholds of < 18.5, 18.5-22.9, 23.0-24.9, and ≥ 25.0 kg/m², respectively [21]. To investigate the effect of body size on AIP and renal function, patients were further dichotomized into underweight/normal weight and overweight/obesity groups using a BMI cutoff of 23 kg/m². Given the BMI distribution of the study population, with a median (interquartile range) BMI of 22.67 (20.53-25.00) kg/m², this cutoff was considered appropriate. Interaction terms were included in fully adjusted models to test heterogeneity. Complete-case analysis using data before imputation was performed as sensitivity analysis to verify robustness of findings.

Two-sided *p*-values < 0.05 were considered statistically significant.

## Results

### Baseline characteristics

**Fig. 2:**
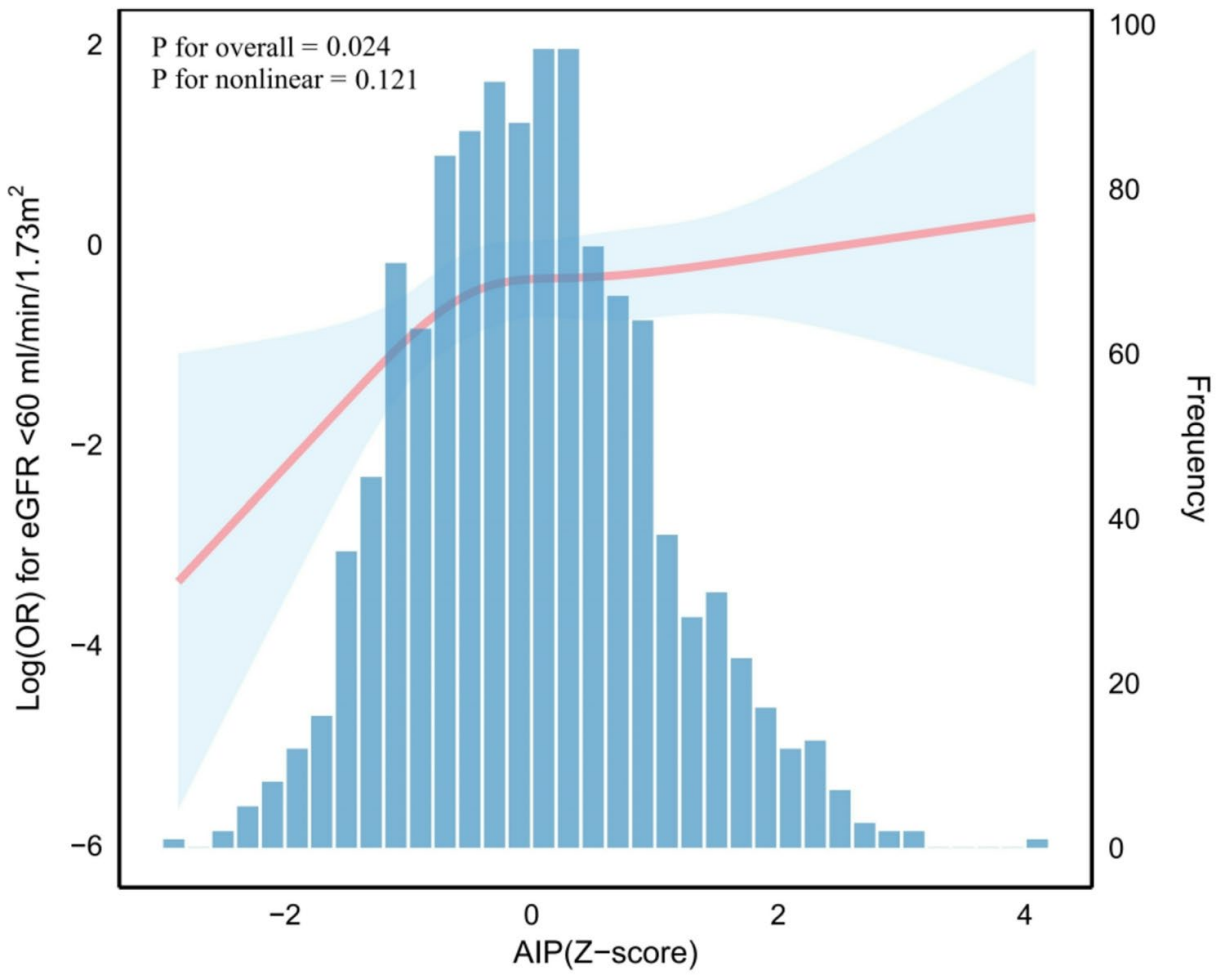
Distribution of AIP Z-scores and their association with eGFR<60 ml/min/1.73 m^2^ in patients with IgA nephropathy. The X-axis represents AIP Z-scores. The left Y-axis displays the log odds ratio for reduced renal function, while the right Y-axis shows the frequency distribution of AIP Z-scores. The deep blue histogram bars illustrate the frequency distribution of AIP Z-scores among 1186 patients with IgA nephropathy. The restricted cubic spline (RCS) analysis (red solid line) demonstrates the relationship between AIP Z-scores and the odds ratio of eGFR<60 ml/min/1.73m^2^. The model was adjusted for age, sex, smoking, BMI, diabetes, SBP, DBP, uric acid, proteinuria, hs-CRP, tubular atrophy/interstitial fibrosis, and statin use. The light blue shaded area surrounding the red line represents the 95% confidence intervals

Among 1186 patients with IgAN, the median age was 34.0 years (IQR: 29.0, 41.0) with 571 (48.2%) males. The distribution of AIP values showed a right-skewed pattern with median 0.05 (IQR: -0.16, 0.26). When standardized as Z-scores, AIP followed a near-normal distribution (Fig. 2). Table 1 presents baseline characteristics stratified by AIP tertiles.

**Table 1:** Baseline demographic and clinical characteristics of IgA nephropathy patients stratified by AIP tertiles

Variables	Total	T1	T2	T3	*P* value
Participants	1186	391	401	394	
Male, n(%)	571 (48.15%)	111 (28.39%)	208 (51.87%)	252 (63.96%)	< 0.001
Age, years	34.00 (29.00, 41.00)	31.00 (27.00, 37.00)	34.00 (29.00, 41.00)	35.00 (31.00, 44.00)	< 0.001
Smoking, n(%)	112 (9.44%)	22 (5.63%)	38 (9.48%)	52 (13.20%)	0.001
Diabetes, n(%)	30 (2.53%)	4 (1.02%)	8 (2.00%)	18 (4.57%)	0.005
BMI, kg/m^2^	22.67 (20.53, 25.00)	20.81 (19.15, 22.77)	22.68 (20.96, 24.66)	24.68 (22.65, 27.06)	< 0.001
SBP, mmHg	125.00 (115.00, 140.00)	120.00 (110.00, 131.00)	127.00 (115.00, 140.00)	130.00 (119.00, 145.00)	< 0.001
DBP, mmHg	82.00 (74.00, 92.00)	79.00 (72.00, 86.00)	83.00 (76.00, 93.00)	85.00 (76.00, 95.00)	< 0.001
WBC, 10^9^/L	6.84 (5.75, 8.03)	6.35 (5.42, 7.47)	6.85 (5.74, 8.10)	7.21 (6.26, 8.45)	< 0.001
Hemoglobin, g/L	127.00 (116.00, 142.00)	122.00 (113.00, 134.00)	128.50 (117.00, 142.00)	133.00 (119.00, 148.00)	< 0.001
Platelets, 10^9^/L	238.00 (200.00, 278.00)	233.50 (196.00, 270.00)	241.00 (198.75, 276.25)	242.50 (205.00, 285.75)	0.019
Albumin, g/L	40.00 (37.00, 42.00)	39.00 (37.00, 42.00)	39.00 (36.00, 42.00)	40.00 (37.00, 43.00)	0.015
FPG, mmol/L	4.59 (4.29, 4.94)	4.45 (4.20, 4.76)	4.63 (4.33, 4.98)	4.75 (4.37, 5.11)	< 0.001
hs-CRP >3 mg/L	131 (11.91%)	21 (5.82%)	50 (13.48%)	60 (16.30%)	< 0.001
TG, mmol/L	1.28 (0.90, 1.85)	0.80 (0.66, 0.95)	1.27 (1.09, 1.47)	2.25 (1.80, 3.10)	< 0.001
TC, mmol/L	4.64 (4.02, 5.40)	4.50 (3.83, 5.15)	4.62 (3.96, 5.25)	4.87 (4.31, 5.73)	< 0.001
HDL-C, mmol/L	1.15 (0.97, 1.37)	1.44 (1.25, 1.66)	1.13 (0.99, 1.27)	0.95 (0.83, 1.09)	< 0.001
LDL-C, mmol/L	2.89 (2.35, 3.47)	2.63 (2.14, 3.16)	2.92 (2.36, 3.46)	3.09 (2.54, 3.82)	< 0.001
IgA, g/L	2.84 (2.26, 3.58)	2.74 (2.18, 3.48)	2.83 (2.29, 3.58)	2.96 (2.34, 3.64)	0.063
IgM, g/L	1.11 (0.81, 1.49)	1.20 (0.88, 1.64)	1.09 (0.79, 1.40)	1.05 (0.80, 1.43)	< 0.001
IgG, g/L	10.54 (8.84, 12.41)	10.81 (9.04, 12.29)	10.55 (8.77, 12.59)	10.31 (8.79, 12.31)	0.554
Complement 3, g/L	1.02 (0.90, 1.16)	0.93 (0.83, 1.05)	1.02 (0.91, 1.16)	1.11 (0.98, 1.24)	< 0.001
Complement 4, g/L	0.23 (0.18, 0.28)	0.20 (0.16, 0.25)	0.23 (0.19, 0.28)	0.26 (0.20, 0.31)	< 0.001
Uric acid, umol/L	398.00 (325.50, 477.00)	352.00 (285.25, 419.75)	399.00 (330.75, 468.00)	448.00 (370.00, 526.00)	< 0.001
eGFR, ml/min/1.73m^2^	86.23 (56.86, 110.64)	101.22 (73.64, 117.88)	84.80 (56.91, 106.66)	73.33 (46.54, 97.22)	< 0.001
CKD stage, n(%)					< 0.001
1	552 (46.54%)	240 (61.38%)	176 (43.89%)	136 (34.52%)	
2	306 (25.80%)	84 (21.48%)	115 (28.68%)	107 (27.16%)	
3	225 (18.97%)	47 (12.02%)	75 (18.70%)	103 (26.14%)	
4	77 (6.49%)	16 (4.09%)	24 (5.99%)	37 (9.39%)	
5	26 (2.19%)	4 (1.02%)	11 (2.74%)	11 (2.79%)	
Proteinuria, mg/24 h	1066.00 (576.00, 1990.00)	818.00 (468.75, 1530.25)	1123.00 (635.00, 1941.50)	1382.50 (752.25, 2623.50)	< 0.001
Oxford Classification, n (%)					
Mesangial hypercellularity (M1)	1010 (85.88%)	332 (85.79%)	345 (86.25%)	333 (85.60%)	0.965
Endocapillary hypercellularity (E1)	303 (25.72%)	99 (25.52%)	94 (23.50%)	110 (28.21%)	0.316
Segmental glomerulosclerosis (S1)	437 (36.97%)	129 (33.16%)	155 (38.65%)	153 (39.03%)	0.163
Tubular atrophy/interstitial (T1/2)	363 (30.81%)	85 (21.91%)	124 (31.08%)	154 (39.39%)	< 0.001
Crescents (C1/2)	615 (52.43%)	193 (50.39%)	210 (52.50%)	212 (54.36%)	0.543
RASi, n (%)	899 (75.80%)	299 (76.47%)	307 (76.56%)	293 (74.37%)	0.718
CSs/ISs, n (%)	500 (43.40%)	161 (42.37%)	176 (45.36%)	163 (42.45%)	0.633
Statin, n (%)	545 (47.47%)	130 (34.39%)	183 (46.92%)	232 (61.05%)	< 0.001
AIP	0.05 (-0.16, 0.26)	-0.25 (-0.34, -0.16)	0.05 (-0.02, 0.12)	0.34 (0.26, 0.52)	< 0.001

Data presented as median (25th, 75th) or number (percent)

*AIP* atherogenic index of plasma, *T* tertile, *BMI* body mass index, *SBP* systolic blood pressure, *DBP* diastolic blood pressure, *WBC* white blood cell, *FPG* fasting plasma glucose, *hs-CRP* high-sensitivity C-reactive protein, *TG* triglyceride, *TC* total cholesterol, *HDL-C* high-density lipoprotein cholesterol, *LDL-C* low-density lipoprotein cholesterol, *IgA* immunoglobulin A, *IgM* immunoglobulin M, *IgG* immunoglobulin G, *eGFR* estimated glomerular filtration rate, *CKD* chronic kidney disease, *RASi* renin-angiotensin system inhibitors, *CSs/ISs* corticosteroids and (or) immunosuppressants

Significant demographic and metabolic gradients were observed across AIP tertiles. Patients in the highest tertile (T3) were more likely to be male (64.0% vs. 28.4% in T1, *p* < 0.001), older (median 35.0 vs. 31.0 years, *p* < 0.001), and had higher prevalence of smoking (13.2% vs. 5.6%, *p* = 0.001) and diabetes (4.6% vs. 1.0%, *p* = 0.005). Metabolic parameters showed progressive deterioration, with BMI increasing from 20.8 kg/m² in T1 to 24.7 kg/m² in T3 (*p* < 0.001). Both systolic (130 vs. 120 mmHg) and diastolic (85 vs. 79 mmHg) blood pressure were significantly higher in T3 compared to T1 (both *p* < 0.001).

Laboratory profiles demonstrated expected lipid patterns across tertiles. TG increased dramatically from 0.80 mmol/L in T1 to 2.25 mmol/L in T3, while HDL-C decreased from 1.44 to 0.95 mmol/L (both *p* < 0.001). TC and LDL-C showed moderate but significant increases from T1 to T3 (TC: 4.50 to 4.87 mmol/L; LDL-C: 2.63 to 3.09 mmol/L; both *p* < 0.001). Other metabolic markers including uric acid, fasting glucose, and inflammatory markers (hs-CRP > 3 mg/L) showed progressive elevation with increasing AIP tertiles.

Regarding medication use, RASi were used by 75.8% of the cohort and CSs/ISs by 43.4%, with no significant differences across AIP tertiles (*p* = 0.718 and *p* = 0.633, respectively). In contrast, statin use increased significantly across tertiles, from 34.4% in T1 to 46.9% in T2 and 61.1% in T3 (*p* < 0.001), consistent with more intensive lipid-lowering therapy among patients with higher AIP values.

Importantly, renal function parameters demonstrated clear inverse associations with AIP levels. Median eGFR decreased from 101.2 mL/min/1.73 m² in T1 to 73.3 mL/min/1.73 m² in T3 (*p* < 0.001), while 24-hour proteinuria increased from 818 to 1383 mg (*p* < 0.001). Among patients with eGFR < 60 mL/min/1.73 m², 67 (17.1%), 110 (27.4%), and 151 (38.3%) were in T1, T2, and T3, respectively. Within the Oxford Classification parameters, only tubular atrophy/interstitial fibrosis showed significant differences across tertiles (21.9% vs. 31.1% vs. 39.4%, *p* < 0.001).

### Association between AIP and renal function

Table 2 presents the association between AIP and eGFR as a continuous outcome. In unadjusted analysis, each standard deviation (SD) increase in AIP was associated with an 8.79 mL/min/1.73 m² decrease in eGFR (95% CI: -10.60, -6.98; *p* < 0.001). This association persisted after adjustment for demographic and clinical variables (Model 2: β: -6.47 mL/min/1.73 m² per SD; 95% CI: -8.38, -4.55; *p* < 0.001) and remained significant in the fully adjusted model (Model 3: β: -2.08 mL/min/1.73 m² per SD; 95% CI: -3.48, -0.68; *p* = 0.004).

**Table 2:** Association between AIP and eGFR in different models (*N* = 1186)

	Model 1	*P* value	Model 2	*P* value	Model 3	*P* value
	β (95% CI)		β (95% CI)		β (95% CI)	
AIP (Z-score)	-8.79 (-10.60, -6.98)	< 0.001	-6.47 (-8.38, -4.55)	< 0.001	-2.08 (-3.48, -0.68)	0.004
Categories						
T1 (*N* = 391)	0 (Ref)		0 (Ref)		0 (Ref)	
T2 (*N* = 401)	-12.92 (-17.35, -8.49)	< 0.001	-8.08 (-12.27, -3.89)	< 0.001	-2.94 (-5.91, -0.03)	0.052
T3 (*N* = 394)	-20.95(-25.40, -16.50)	< 0.001	-15.05 (-19.65, -10.45)	< 0.001	-4.73 (-8.06, -1.41)	0.005
P for trend	< 0.001		< 0.001		0.005	

Model 1: unadjusted

Model 2: adjusted for age, sex, smoking, BMI, diabetic, SBP, DBP

Model 3: adjusted for model 2 covariates and UA, proteinuria, hs-CRP, tubular atrophy/interstitial fibrosis, and statin use

*AIP* atherogenic index of plasma, *β* beta coefficient, *CI* confidence interval, *T* tertile, *Ref* reference

Categorical analysis by tertiles revealed a dose-response relationship. In the fully adjusted model, compared to T1, patients in T2 had 2.94 mL/min/1.73 m² lower eGFR (95% CI: -5.91, -0.03; *p* = 0.052), while those in T3 exhibited 4.73 mL/min/1.73 m² lower eGFR (95% CI: -8.06, -1.41; *p* = 0.005). The *p*-value for trend across tertiles was 0.005.

Table 3 shows associations with eGFR < 60 mL/min/1.73 m². Each SD increase in AIP conferred 57% higher odds of eGFR < 60 mL/min/1.73 m² in unadjusted analysis (OR: 1.57; 95% CI: 1.37, 1.79; *p* < 0.001). This association remained robust after progressive adjustments, with fully adjusted OR of 1.33 (95% CI: 1.07, 1.67; *p* = 0.012). Patients in T3 had 3-fold higher odds of eGFR < 60 mL/min/1.73 m² compared to T1 in unadjusted analysis, which attenuated but remained significant after full adjustment (OR: 1.76; 95% CI: 1.03, 3.00; *p* = 0.034).

**Table 3:** Association between AIP and eGFR<60 ml/min/1.73m^2^ in different models (*N* = 1186)

	Model 1	*P* value	Model 2	*P* value	Model 3	*P* value
	OR (95% CI)		OR (95% CI)		OR (95% CI)	
AIP (Z-score)	1.57 (1.37, 1.79)	< 0.001	1.58(1.34, 1.87)	< 0.001	1.33 (1.07, 1.67)	0.012
Categories						
T1 (*N* = 391)	1.00 (Ref )		1.00 (Ref )		1.00 (Ref )	
T2 (*N* = 401)	1.83 (1.30, 2.58)	< 0.001	1.59 (1.08, 2.32)	0.018	1.19 (0.71, 1.98)	0.512
T3 (*N* = 394)	3.00 (2.15, 4.19)	< 0.001	2.81(1.88, 4.19)	< 0.001	1.76 (1.03, 3.00)	0.034
P for trend	< 0.001		< 0.001		0.029	

There were 67, 110, and 151 participants with eGFR < 60 ml/min/1.73 m² in the T1, T2, and T3 groups, respectively

Model 1: Unadjusted

Model 2: Adjusted for age, sex, smoking, BMI, diabetic, SBP, DBP

Model 3: Adjusted for model 2 covariates and UA, proteinuria, hs-CRP, tubular atrophy/interstitial fibrosis, and statin use

*AIP* atherogenic index of plasma, *OR* odds ratio, *CI* confidence interval, *T* tertile, *Ref* reference

The RCS analysis confirmed a linear increase in eGFR < 60 mL/min/1.73 m² risk as AIP (Z-score) rose. The overall association was significant (p for overall = 0.024), with no evidence of non-linearity (p for nonlinear = 0.121), as shown in Fig. 2.

### Incremental value of AIP beyond established predictors

To further evaluate whether AIP provides incremental explanatory information beyond established clinical, laboratory, and histological predictors, we compared model performance with and without AIP. The baseline model included the established predictors specified in Model 3 of the primary analyses.

For eGFR as a continuous outcome, inclusion of AIP modestly improved model performance, as indicated by a higher mean R² (0.632 vs. 0.635; Δ = 0.003) and a lower mean AIC (10493.9 vs. 10487.1). Likelihood ratio testing consistently demonstrated that adding AIP significantly improved model fit (mean *p* = 0.004).

For the binary outcome of eGFR < 60 mL/min/1.73 m², adding AIP resulted in a small increase in mean AUC (0.918 vs. 0.920; Δ = 0.002); however, this improvement did not reach statistical significance by DeLong test (mean *p* = 0.094).

### Association between AIP and proteinuria

We examined the relationship between AIP and 24-hour proteinuria levels (Table S3). In unadjusted analysis, each SD increase in AIP was associated with 326.01 mg/24 h higher proteinuria (95% CI: 224.73, 427.29; *p* < 0.001). After adjustment for demographic and clinical factors (Model 2), this association remained significant (β: 217.61 mg/24 h per SD; 95% CI: 102.55, 332.66; *p* < 0.001). However, in the fully adjusted model (Model 3), the association was attenuated and no longer statistically significant (β: 73.00 mg/24 h per SD; 95% CI: -37.68, 183.69; *p* = 0.196).

Stratified analyses by renal function revealed heterogeneous associations. Among patients with eGFR ≥ 60 mL/min/1.73 m² (*n* = 858), AIP remained significantly associated with proteinuria when analyzed as a continuous variable across all models, including the fully adjusted model (Model 3; β: 141.63 mg/24 h per SD; 95% CI: 33.75, 249.51; *p* = 0.01). In tertile analyses, the associations were progressively attenuated with additional adjustment; although the highest tertile showed a higher point estimate of proteinuria compared with the lowest tertile in Model 3 (β: 221.04 mg/24 h), this difference did not reach statistical significance (95% CI: -38.74, 480.81; *p* = 0.095), and no significant linear trend was observed (p for trend = 0.098) (Table S4). In contrast, among patients with eGFR < 60 mL/min/1.73 m² (*n* = 328), AIP was not significantly associated with proteinuria in any model, including the fully adjusted model (β: -84.46 mg/24 h per SD; 95% CI: -369.68, 200.77; *p* = 0.561) (Table S5).

### Subgroup and sensitivity analyses

**Fig. 3:**
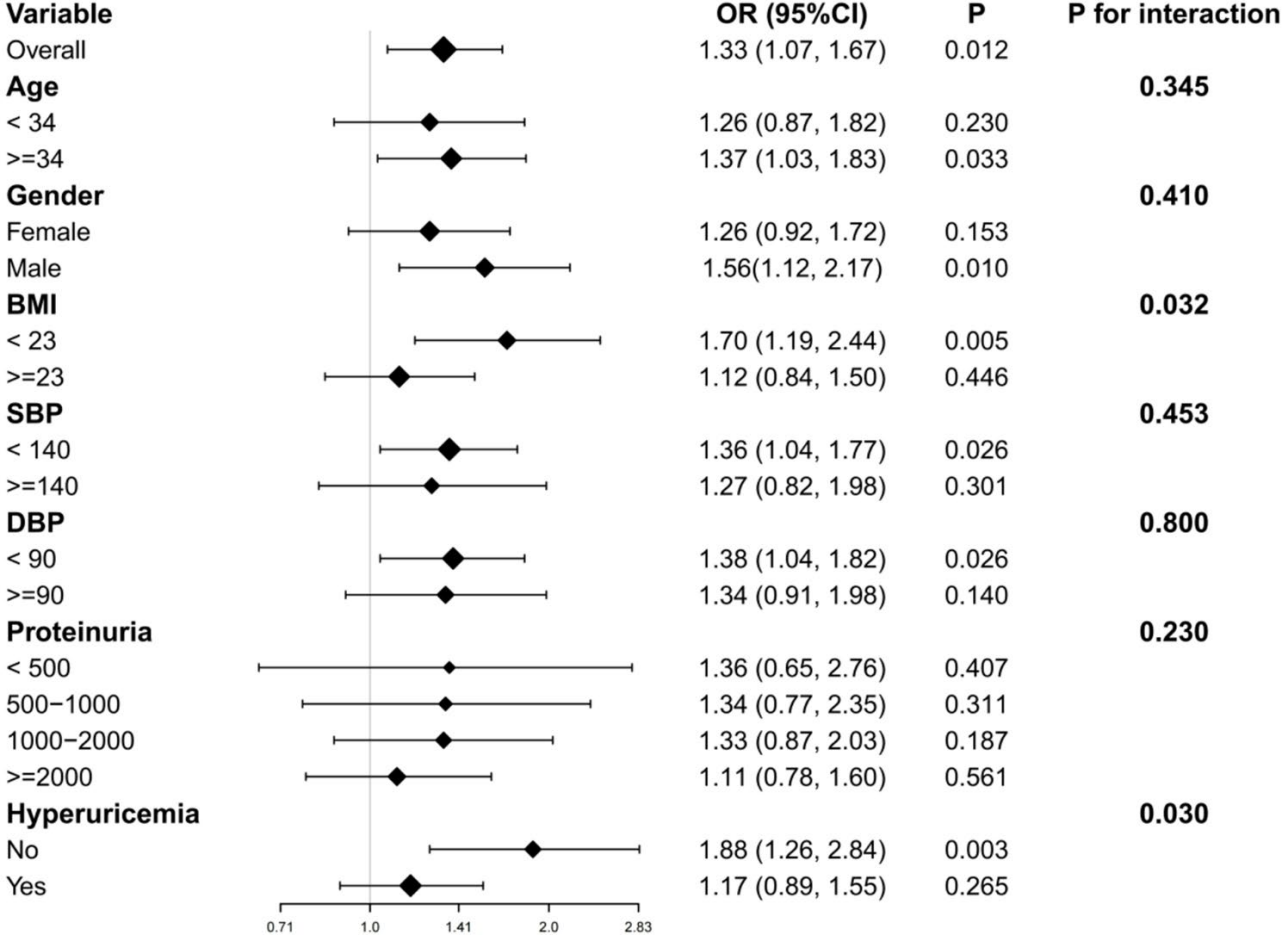
Subgroup and interaction analyses of the association between AIP Z-scores and eGFR<60 ml/min/1.73 m^2^ in patients with IgA nephropathy. The models were adjusted for age, sex, smoking, BMI, diabetes, SBP, DBP, uric acid, proteinuria, hs-CRP, tubular atrophy/interstitial fibrosis, and statin use. For each subgroup analysis, the stratification variable was excluded from the adjustment covariates to prevent over-adjustment. Interaction terms were introduced into the fully adjusted models to evaluate the heterogeneity of associations across different subgroups

Subgroup analyses revealed significant effect modification by metabolic factors (Fig. 3). The association between AIP and eGFR < 60 mL/min/1.73 m² was significantly stronger in patients with BMI < 23 kg/m² (OR: 1.70; 95% CI: 1.19, 2.44; *p* = 0.005) compared to those with BMI ≥ 23 kg/m² (OR: 1.12; 95% CI: 0.84, 1.50; *p* = 0.446) (*p*-interaction = 0.032). Similarly, patients without hyperuricemia showed stronger associations (OR: 1.88; 95% CI: 1.26, 2.84; *p* = 0.003) than those with hyperuricemia (OR: 1.17; 95% CI: 0.89, 1.55; *p* = 0.265) (*p*-interaction = 0.030).

Sensitivity analyses using complete-case data (without imputation, Tables S6-S7) confirmed the robustness of our findings. In the fully adjusted complete-case analysis (*n* = 968), the association between AIP and eGFR (β: -2.74 mL/min/1.73 m² per SD; 95% CI: -4.33, -1.14; *p* < 0.001) and the association with eGFR < 60 mL/min/1.73 m² (OR: 1.51; 95% CI: 1.17, 1.96; *p* = 0.002) remained statistically significant and consistent with the results from our primary analyses using multiply imputed data. The dose-response relationship was also preserved, with patients in the highest AIP tertile showing significantly lower eGFR (β: -5.84 mL/min/1.73 m² per SD; 95% CI: -9.61, -2.07; *p* = 0.002) and higher odds of eGFR < 60 mL/min/1.73 m² (OR: 2.24 ; 95% CI: 1.23, 4.15; *p* = 0.009) compared to those in the lowest tertile.

## Discussion

This cross-sectional study of 1186 Chinese patients with IgAN demonstrates that elevated AIP is independently associated with lower eGFR. After multivariable adjustment, each SD increase in AIP was associated with 2.08 mL/min/1.73 m² lower eGFR and 33% higher odds of eGFR < 60 mL/min/1.73 m². A robust dose-response was observed: patients in the highest AIP tertile had 4.73 mL/min/1.73 m² lower eGFR and 76% higher odds of eGFR < 60 mL/min/1.73 m² compared with the lowest tertile. AIP was associated with higher proteinuria only in patients with eGFR ≥ 60 mL/min/1.73 m² (β: 141.63 mg/24 h per SD). Effect modification by BMI and hyperuricemia was significant (*p*-interaction = 0.032 and 0.030), with stronger associations in patients with BMI < 23 kg/m² and without hyperuricemia. Findings were consistent in complete-case analyses. Collectively, these findings demonstrate that AIP is independently associated with renal function and proteinuria at diagnosis in IgAN, with associations modified by metabolic factors, suggesting that dyslipidemia may play a role in disease presentation.

In general populations, prior studies have shown that lipid indices predict renal injury, with TG/HDL-C predicting albuminuria and values below a threshold of 0.91 associated with higher microalbuminuria risk [12, 13]. In IgAN cohorts, dyslipidemia portends worse outcomes: hypertriglyceridemia accelerated progression to renal endpoints among patients with advanced glomerulosclerosis (47.4% compared with 25.2% without hypertriglyceridemia) [22], and TG/HDL-C ≥ 1.495 independently predicted end-stage renal disease (ESRD), particularly in those with lower baseline eGFR [23]. Using AIP, a log transformed TG/HDL-C index, we modeled risk continuously rather than by cutoffs, showing a consistent, independent dose-response association across IgAN severity after multivariable adjustment. Notably, renal dysfunction itself is known to induce a characteristic dyslipidemia marked by hypertriglyceridemia, reduced HDL-C, and impaired lipoprotein clearance. This raises the possibility that elevated AIP may, at least in part, reflect declining renal function rather than act as an upstream driver of kidney injury. The strong gradient of CKD stage across AIP tertiles in our cohort further supports this concern and underscores the challenge of disentangling cause from consequence in cross-sectional analyses. The association with higher proteinuria, especially in early-stage disease, supports dyslipidemia’s contribution to glomerular and tubular injury via multiple pathways. Together with effect modification by BMI and hyperuricemia, this mathematical amplification of atherogenic balance suggests AIP captures the metabolic dimension of disease severity at presentation in IgAN. Whether serial AIP measurements can monitor disease progression or guide therapeutic decisions requires prospective investigation.

Effect modification by metabolic factors underscores heterogeneity in the IgAN cohort. Associations were stronger in patients with BMI below 23 kg/m² (OR 1.70 for eGFR < 60 mL/min/1.73 m²) and in those without hyperuricemia (OR 1.88), suggesting that AIP may be more informative in the absence of overt metabolic comorbidity. However, these subgroup findings should be interpreted cautiously, as they are based on exploratory interaction analyses in a cross-sectional study and may be influenced by multiple testing and residual confounding. Notably, these observations raise the possibility that AIP may identify metabolically vulnerable patients who are not captured by BMI- or uric acid-based stratification. Epidemiological data support this: a systematic review and meta-analysis by Thomas et al. reported that elevated triglycerides and low HDL-C are independently associated with incident CKD and highlighted BMI’s limitations, with central adiposity outperforming BMI and adverse profiles evident even among ‘lean’ individuals [24]. Although that work did not assess AIP or BMI-stratified interactions, it supports the premise that lipid-based indices can reveal renal risk not captured by BMI, consistent with stronger associations at lower BMI. Conversely, in patients with more advanced CKD or established metabolic abnormalities, the observed attenuation of associations may reflect a combination of treatment effects, residual confounding, and reverse causality driven by renal function-dependent lipid alterations, underscoring the need for comprehensive metabolic profiling rather than isolated lipid metrics [24].

While our cross-sectional design precludes mechanistic conclusions, the preferential association of AIP with tubular atrophy/interstitial fibrosis (39.4% in the highest tertile compared with 21.9% in the lowest) aligns with known pathophysiology. Experimental data link renal lipid accumulation to kidney injury and fibrosis [25], and in tubular cells, impaired fatty-acid oxidation promotes toxic lipid intermediates via transforming growth factor-beta 1(TGF-β1) signaling [26]. The renal function-dependent association with proteinuria further supports this framework: among patients with preserved renal function, the association may reflect early glomerular lipotoxic injury, whereas in established dysfunction, competing processes may obscure this relationship. In IgAN, aberrant IgA1 glycosylation [16] plus metabolic burden may synergistically accelerate progression. Mendelian randomization linking genetically higher triglycerides to IgAN risk provides genetic support [27].

Our findings highlight the metabolic dimension of IgAN at presentation. As a readily available and cost-free index derived from routine lipid profiles, AIP demonstrated a robust dose-response relationship with renal function that was independent of traditional clinical and histologic risk factors. Importantly, the potential value of AIP lies not in replacing established predictors such as eGFR, proteinuria, or the Oxford classification, but in complementing them by capturing metabolic burden not fully reflected by conventional markers. In our analyses, the association between AIP and renal function persisted after comprehensive adjustment, suggesting that AIP may provide incremental information regarding disease severity at diagnosis. Furthermore, exploratory model comparison analyses showed that adding AIP significantly improved overall model fit for continuous eGFR (lower AIC and significant likelihood ratio test), whereas the improvement in discrimination for predicting eGFR < 60 mL/min/1.73 m² was small and not statistically significant by DeLong test, likely owing to the already strong performance of the baseline model. Clinically, AIP may therefore help refine risk stratification, particularly in patients without overt metabolic comorbidities, by identifying individuals with disproportionate metabolic stress who may warrant closer monitoring or targeted management of modifiable metabolic factors. However, whether incorporation of AIP into routine assessment improves prognostic discrimination beyond existing models or influences clinical decision-making cannot be determined from this cross-sectional study. Although current KDIGO guidelines do not prioritize triglyceride reduction or HDL-C elevation [9], our findings suggest that dyslipidemia warrants attention in IgAN. In this context, AIP may serve as an integrated marker of metabolic stress accompanying renal impairment rather than a causal risk factor per se, and longitudinal studies are required to clarify its temporal and prognostic relevance.

Several limitations warrant consideration. First, the cross-sectional design precludes causal inference and raises the possibility of reverse causality, given the bidirectional relationship between dyslipidemia and kidney dysfunction. As all measurements were obtained at the time of renal biopsy, it remains unclear whether elevated AIP precedes renal function decline, results from reduced eGFR, or simply reflects disease severity at presentation. The lack of longitudinal follow-up further limits assessment of AIP as an independent predictor of renal progression. Although statin use was adjusted for, detailed information on the duration and intensity of lipid-lowering therapy was unavailable, and data on dietary habits and physical activity were not collected. In addition, despite the large sample size, the single-center design may limit generalizability, as regional differences in genetic background, environmental exposures, and clinical practice patterns could influence the applicability of our findings to other IgAN populations. Finally, the observed effect modification by BMI and hyperuricemia should be regarded as hypothesis-generating and requires confirmation in independent cohorts with longitudinal follow-up. Prospective multicenter studies incorporating serial AIP measurements are warranted to clarify its prognostic value and the underlying lipid-mediated mechanisms in IgAN.

## Conclusions

This study demonstrates that AIP exhibits significant inverse associations with renal function in Chinese IgAN patients, with effect modification revealing particularly vulnerable subgroups. While these findings support incorporating comprehensive lipid assessment beyond traditional parameters in IgAN evaluation, prospective studies are essential to determine whether AIP predicts long-term renal outcomes and whether AIP-guided interventions can improve prognosis in this prevalent glomerulopathy.

## Supplementary Information

Supplementary Material 1.

## Data Availability

The datasets used and analyzed during the current study are available from the corresponding author on reasonable request.

## Abbreviations

AIP: Atherogenic index of plasma
β: Beta coefficient
BMI: Body mass index
CI: Confidence interval
CKD: Chronic kidney disease
CKD-EPI: Chronic kidney disease epidemiology collaboration
CSs/ISs: Corticosteroids and/or immunosuppressants
DBP: Diastolic blood pressure
eGFR: Estimated glomerular filtration rate
ESRD: End-stage renal disease
FPG: Fasting plasma glucose
HDL-C: High-density lipoprotein cholesterol
Hs-CRP: High-sensitivity C-reactive protein
IgAN: IgA nephropathy
IgA: Immunoglobulin A
IgM: Immunoglobulin M
IgG: Immunoglobulin G
IQR: Interquartile range
KDIGO: Kidney Disease: Improving Global Outcomes
LDL-C: Low-density lipoprotein cholesterol
OR: Odds ratio
RCS: Restricted cubic spline
RASi: Renin-angiotensin system inhibitors
Ref: Reference
SBP: Systolic blood pressure
SD: Standard deviation
T: Tertile
TC: Total cholesterol
TG: Triglycerides
TGF-β1: Transforming growth factor-beta 1
WBC: White blood cell
