# Supplementary material for: Atherogenic index of plasma and lower estimated glomerular filtration rate in IgA nephropathy
Source: Lipids Health Dis. 2026 Feb 18;25:69. doi: 10.1186/s12944-026-02896-4 (PMC12934030; doi:10.1186/s12944-026-02896-4)
Supplement: Supplementary file 1 — Supplementary Material 1. [file 12944_2026_2896_MOESM1_ESM.docx]

**Supplementary Material**

**Table S1.** Test of normality

| **Kolmogorov-Smirnov** | **Statistic** | **P value** |
| --- | --- | --- |
| Age, years | 0.115 | <0.001 |
| BMI, kg/m^2^ | 0.052 | <0.001 |
| SBP, mmHg | 0.078 | <0.001 |
| DBP, mmHg | 0.072 | <0.001 |
| WBC, 10^9^/L | 0.072 | <0.001 |
| Hemoglobin, g/L | 0.033 | 0.004 |
| Platelets, 10^9^/L | 0.051 | <0.001 |
| Albumin, g/L | 0.100 | <0.001 |
| FPG, mmol/L | 0.087 | <0.001 |
| TG, mmol/L | 0.165 | <0.001 |
| TC, mmol/L | 0.083 | <0.001 |
| HDL-C，mmol/L | 0.086 | <0.001 |
| LDL-C，mmol/L | 0.072 | <0.001 |
| IgA, g/L | 0.074 | <0.001 |
| IgM, g/L | 0.100 | <0.001 |
| IgG, g/L | 0.057 | <0.001 |
| Complement 3, g/L | 0.042 | <0.001 |
| Complement 4, g/L | 0.168 | <0.001 |
| Uric acid, umol/L | 0.044 | <0.001 |
| eGFR, ml/min/1.73m^2^ | 0.076 | <0.001 |
| Proteinuria, mg/24h | 0.187 | <0.001 |
| AIP | 0.031 | 0.008 |

AIP: atherogenic index of plasma; BMI: body mass index; SBP: systolic blood pressure; DBP: diastolic blood pressure; WBC: white blood cell; FPG: fasting plasma glucose; TG: triglyceride; TC: total cholesterol; HDL-C: high-density lipoprotein cholesterol; LDL-C: low-density lipoprotein cholesterol; IgA: immunoglobulin A; IgM: immunoglobulin M; IgG: immunoglobulin G; eGFR: estimated glomerular filtration rate.

**Table S2.** Distribution of variables with missing data

| **Variables** | **Missing (%)** |
| --- | --- |
| BMI | 124 (10.46%) |
| FPG | 74 (6.24%) |
| hs-CRP | 86 (7.25%) |
| IgA | 50 (4.22%) |
| IgM | 53 (4.47%) |
| IgG | 55 (4.64%) |
| Complement 3 | 50 ( 4.22%) |
| Complement 4 | 51 (4.30%) |
| Uric acid | 3 (0.25%) |
| Proteinuria | 5 (0.42%) |
| Mesangial hypercellularity (M) | 10 (0.84%) |
| Intracapillary proliferation (E) | 8 (0.67%) |
| Segmental glomerulosclerosis (S) | 4 (0.34%) |
| tubular atrophy/interstitial fibrosis (T) | 8 (0.67%) |
| Crescents (C) | 13 (1.10%) |
| CSs/ISs | 34 (2.87%) |
| Statin | 38 (3.20%) |

BMI: body mass index; FPG: fasting plasma glucose; hs-CRP: high-sensitivity C-reactive protein; IgA: immunoglobulin A; IgM: immunoglobulin M; IgG: immunoglobulin G; CSs/ISs: corticosteroids and (or) immunosuppressants.

**Table S3.** Association between AIP and proteinuria in different models (N=1186)

|  | **Model 1** | P value | **Model 2** | P value | **Model 3** | P value |
| --- | --- | --- | --- | --- | --- | --- |
|  | **β (95% CI)** |  | **β (95% CI)** |  | **β (95% CI)** |  |
| AIP  (Z-score) | 326.01  (224.73, 427.29) | <0.001 | 217.61  (102.55, 332.66) | <0.001 | 73.00  ( -37.68, 183.69) | 0.196 |
| Categories |  |  |  |  |  |  |
| T1 (N=391) | 0 (Ref) |  | 0 (Ref) |  | 0 (Ref) |  |
| T2 (N=401) | 374.21  (125.15,623.27) | 0.003 | 205.20  (-46.73, 457.12) | 0.110 | 5.50  (-230.19,241.18) | 0.964 |
| T3 (N=394) | 745.60  (495.99, 995.21) | <0.001 | 447.17  (170.58,723.76) | 0.002 | 93.93  (-170.32, 358.18) | 0.486 |
| P for trend | <0.001 |  | 0.002 |  | 0.481 |  |

Model 1: unadjusted.

Model 2: adjusted for age, sex, smoking, BMI, diabetic, SBP, DBP.

Model 3: adjusted for model 2 covariates and UA, eGFR, hs-CRP, tubular atrophy/interstitial fibrosis, and statin use.

AIP: atherogenic index of plasma; β: beta coefficient; CI: confidence interval; T: tertile; Ref: reference.

|  | **Model 1** | P value | **Model 2** | P value | **Model 3** | P value |
| --- | --- | --- | --- | --- | --- | --- |
|  | **β (95% CI)** |  | **β (95% CI)** |  | **β (95% CI)** |  |
| AIP  (Z-score) | 229.35  (140.27, 318.43) | <0.001 | 201.61  ( 94.83,308.38) | <0.001 | 141.63  (33.75, 249.51) | 0.01 |
| Categories |  |  |  |  |  |  |
| T1  (N=324) | 0 (Ref) |  | 0 (Ref) |  | 0 (Ref) |  |
| T2  (N=291) | 235.60  (22.54,448.65) | 0.03 | 191.85  (-30.84,414.55) | 0.091 | 76.12  (-143.96, 296.20) | 0.497 |
| T3  (N=243) | 495.45  (271.62, 719.29) | <0.001 | 374.84  (116.21,633.47) | 0.005 | 221.04  (-38.74, 480.81) | 0.095 |
| P for trend | <0.001 |  | 0.004 |  | 0.098 |  |

**Table S4.** Association between AIP and proteinuria in patients with eGFR≥60ml/min/1.73m^2^ in different models (N=858)

Model 1: unadjusted.

Model 2: adjusted for age, sex, smoking, BMI, diabetic, SBP, DBP.

Model 3: adjusted for model 2 covariates and UA, eGFR, hs-CRP, tubular atrophy/interstitial fibrosis, and statin use.

AIP: atherogenic index of plasma; β: beta coefficient; CI: confidence interval; T: tertile; Ref: reference.

**Table S5.** Association between AIP and proteinuria in patients with eGFR＜60ml/min/1.73m^2^ in different models (N=328)

|  | **Model 1** | P value | **Model 2** | P value | **Model 3** | P value |
| --- | --- | --- | --- | --- | --- | --- |
|  | **β (95% CI)** |  | **β (95% CI)** |  | **β (95% CI)** |  |
| AIP  (Z-score) | 113.88  (-165.04,392.81) | 0.422 | -45.39  (-338.53,247.74) | 0.761 | -84.46  (-369.68, 200.77) | 0.561 |
| Categories |  |  |  |  |  |  |
| T1  (N=67) | 0 (Ref) |  | 0 (Ref) |  | 0 (Ref) |  |
| T2  (N=110) | 180.35  (-531.88, 892.58) | 0.619 | -23.89  (-718.20, 670.43) | 0.946 | -217.29  (-892.91, 458.32) | 0.527 |
| T3  (N=151) | 322.41  (-350.15,994.98) | 0.346 | -69.38  ( -770.92,632.16) | 0.846 | -306.24  (-993.10, 380.63) | 0.381 |
| P for trend | 0.340 |  | 0.835 |  | 0.398 |  |

Model 1: unadjusted.

Model 2: adjusted for age, sex, smoking, BMI, diabetic, SBP, DBP.

Model 3: adjusted for model 2 covariates and UA, eGFR, hs-CRP, tubular atrophy/interstitial fibrosis, and statin use.

AIP: atherogenic index of plasma; β: beta coefficient; CI: confidence interval; T: tertile; Ref: reference.

**Table S6.** Association between AIP and eGFR in different models with unimputed data

|  | **Model 1 (n=1186)** | P value | **Model 2 (n=1062)** | P value | **Model 3 (n=968)** | P value |
| --- | --- | --- | --- | --- | --- | --- |
|  | **β (95% CI)** |  | **β (95% CI)** |  | **β (95% CI)** |  |
| AIP  (Z-score) | -8.84 (-10.66, -7.03) | <0.001 | -6.33 (-8.38, -4.29) | <0.001 | -2.74 (-4.33, -1.14) | <0.001 |
| Categories |  |  |  |  |  |  |
| T1 | 0 (Ref) |  | 0 (Ref) |  | 0 (Ref) |  |
| T2 | -12.92 (-17.35, -8.50) | <0.001 | -8.22 (-12.63, -3.81) | <0.001 | -3.32 (-6.65, 0.02) | 0.051 |
| T3 | -20.95(-25.40, -16.51) | <0.001 | -14.92 (-19.84, -10.00) | <0.001 | -5.84 (-9.61, -2.07) | 0.002 |
| P for trend | <0.001 |  | <0.001 |  | 0.002 |  |

Model 1: unadjusted.

Model 2: adjusted for age, sex, smoking, BMI, diabetic, SBP, DBP.

Model 3: adjusted for model 2 covariates and UA, proteinuria, hs-CRP, tubular atrophy/interstitial fibrosis, and statin use.

AIP: atherogenic index of plasma; β: beta coefficient; CI: confidence interval; T: tertile; Ref: reference.

**Table S7.** Association between AIP and eGFR＜60ml/min/1.73m^2^ in different models with unimputed data

|  | **Model 1 (n=1186)** | P value | **Model 2 (n=1062)** | P value | **Model 3 (n=968)** | P value |
| --- | --- | --- | --- | --- | --- | --- |
|  | **OR (95% CI)** |  | **OR (95% CI)** |  | **OR (95% CI)** |  |
| AIP  (Z-score) | 1.57 (1.38, 1.79) | <0.001 | 1.58(1.33, 1.89) | <0.001 | 1.51 (1.17, 1.96) | 0.002 |
| Categories |  |  |  |  |  |  |
| T1 | 1.00 (Ref ) |  | 1.00 (Ref ) |  | 1.00 (Ref ) |  |
| T2 | 1.83 (1.30, 2.57) | <0.001 | 1.52 (1.02, 2.27) | 0.040 | 1.17 (0.66, 2.07) | 0.597 |
| T3 | 3.00 (2.16, 4.19) | <0.001 | 2.82 (1.84, 4.31) | <0.001 | 2.24 (1.23, 4.15) | 0.009 |
| P for trend | <0.001 |  | <0.001 |  | 0.006 |  |

Model 1: unadjusted.

Model 2: adjusted for age, sex, smoking, BMI, diabetic, SBP, DBP.

Model 3: adjusted for model 2 covariates and UA, proteinuria, hs-CRP, tubular atrophy/interstitial fibrosis, and statin use.

AIP: atherogenic index of plasma; OR: odds ratio; CI: confidence interval; T: tertile; Ref: reference.
